# RNF185, a Novel Mitochondrial Ubiquitin E3 Ligase, Regulates Autophagy through Interaction with BNIP1

**DOI:** 10.1371/journal.pone.0024367

**Published:** 2011-09-09

**Authors:** Fei Tang, Bin Wang, Na Li, Yanfang Wu, Junying Jia, Talin Suo, Quan Chen, Yong-Jun Liu, Jie Tang

**Affiliations:** 1 Key Laboratory of Infection and Immunity, Institute of Biophysics, Chinese Academy of Sciences, Beijing, China; 2 State Key Laboratory of Biomembrane and Membrane Biotechnology, Institute of Zoology, Chinese Academy of Sciences, Beijing, China; 3 Graduate University of Chinese Academy of Sciences, Beijing, China; 4 Department of Immunology and Center for Cancer Immunology Research, University of Texas MD Anderson Cancer Center, Houston, Texas, United States of America; Chinese University of Hong Kong, Hong Kong

## Abstract

Autophagy is an evolutionarily conserved catabolic process that allows recycling of cytoplasmic organelles, such as mitochondria, to offer a bioenergetically efficient pathway for cell survival. Considerable progress has been made in characterizing mitochondrial autophagy. However, the dedicated ubiquitin E3 ligases targeting mitochondria for autophagy have not been revealed. Here we show that human RNF185 is a mitochondrial ubiquitin E3 ligase that regulates selective mitochondrial autophagy in cultured cells. The two C-terminal transmembrane domains of human RNF185 mediate its localization to mitochondrial outer membrane. RNF185 stimulates LC3II accumulation and the formation of autophagolysosomes in human cell lines. We further identified the Bcl-2 family protein BNIP1 as one of the substrates for RNF185. Human BNIP1 colocalizes with RNF185 at mitochondria and is polyubiquitinated by RNF185 through K63-based ubiquitin linkage *in vivo*. The polyubiquitinated BNIP1 is capable of recruiting autophagy receptor p62, which simultaneously binds both ubiquitin and LC3 to link ubiquitination and autophagy. Our study might reveal a novel RNF185-mediated mechanism for modulating mitochondrial homeostasis through autophagy.

## Introduction

The ubiquitin proteasome system (UPS) is well known to be involved in diverse cellular processes, including development, proliferation, transcription, signal transduction, apoptosis, and DNA repair[1], [2], [3], [4]. Ubiquitin E3 ligases play central regulatory roles of UPS in that they provide substrate specificity and catalyze the ligation of ubiquitin to the substrate. Our understanding of E3 ligases has been improved dramatically with the discovery of the RING (Really Interesting New Gene) domain as a module for E3 ligases. The RING domain comprises eight cysteine and histidine residues together (such as C3HC4) that bind two atoms of zinc to form one unique cross-braced minidomain, yielding a rigid, globular platform for protein-protein interactions. RING domain proteins are comprising >95% of all predicted human E3 ligases[5], implying a very broad involvement of RING-dependent ubiquitination *in vivo* [6], [7].

Ubiquitination plays the important regulatory role mainly targeting substrates for degradation by the 26S proteasome. However, the proteasome is limited in its capacity for degrading individual proteins. Removal of aggregated proteins, larger macromolecular complexes and whole organelles is mediated by autophagy, a catabolic process in which cytosolic cellular components are delivered to the lysosome for degradation. Ubiquitination has been proposed as a signal for selective autophagy[8], and the autophagy receptor proteins, such as p62 and NBR1, interact with both ubiquitin and autophagosome-specific Atg8-family proteins LC3 (microtubule-associated protein light chain 3)/GABARAP, to promote autophagy[9].

The role of autophagy in the control of mitochondrial degradation is now generally recognized[10], [11], [12], [13]. The autophagic uptake of mitochondria and their subsequent degradation in lysosome accentuates the importance of mitochondrial degradation by autophagy for cellular homeostasis. However, how mitochondria are selected for degradation by autophagy remains largely unknown. The removal of mitochondria can be specific, and the signals that specify mitochondria as targets of the autophagical process have recently begun to be elucidated both in yeast and mammalian cells [14], [15], [16], [17]. In mammalian cells, the activation of mitochondrial permeability transition and loss of mitochondrial membrane potential appear to be common features of mitochondrial autophagy[18]. The reactive oxygen species (ROS) of mitochondrial origin are also proposed as signaling molecules for mitochondrial autophagy regulation[19], [20]. Bif-1 is involved in the regulation of mitochondria autophagy by stimulating Bax and interacting with Beclin 1 through UVRAG[21], [22]. The fission/fussion machinery of mitochondria has also been associated with autophagy[11], [23], although direct involvement has not been demonstrated.

Despite the considerable progress in characterizing mitochondrial autophagy, relatively little is known about the genes that regulate selective autophagy of mitochondria through ubiquitination. Meanwhile, the study of ubiquitination system in mitochondrial biology is still at an early stage as only a few mitochondria related E3 ligases are known. Here, we describe the identification of a novel RING domain protein RNF185 as a mitochondrial outer membrane (MOM) ubiquitin E3 ligase involved in the regulation of selective autophagy.

## Results

### RNF185 localizes at mitochondria

**Figure 1:**
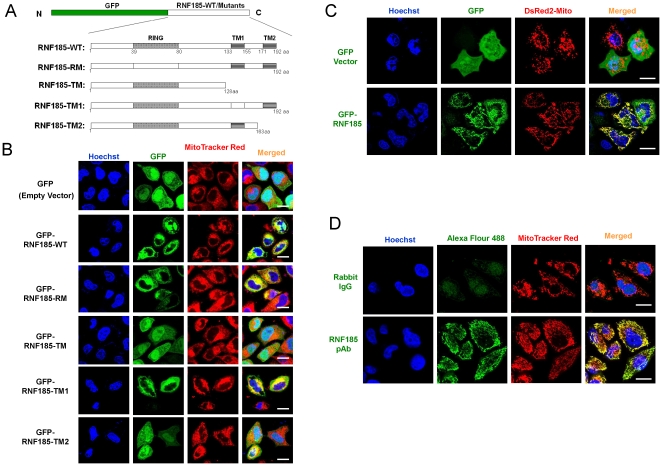
Mitochondrial localization of exogenously expressed and endogenous RNF185. (**A**) schematic presentation of GFP tagged full-length RNF185 and its mutants. RNF185-WT, wild type RNF185; RNF185-RM, RING domain mutated; RNF185-TM, two TM domains deleted; RNF185-TM1, the first TM domain mutated; RNF185-TM2, the second TM domain deleted. (**B**) RNF185's predicted TM domains mediated its localization to mitochondria. GFP tagged wild type or mutated RNF185 was transfected into HeLa cells and MitoTracker Red was used to stain mitochondria at 24 h post transfection. (**C**) GFP-RNF185 colocalized with DsRed2-Mito. Plasmid encoding GFP tagged wild type RNF185 (GFP-RNF185) and pDsRed2-Mito were co-transfected into HeLa cells and confocal microscopic analysis was taken at 24 h post transfection. (**D**) Endogenous RNF185 localized at mitochondria. HeLa cells were subjected to immunocytochemistry-based confocal microscopic analysis with Rabbit IgG or affinity chromatography purified polyclonal antibody (pAb) raised against RNF185. Alexa fluor 488-conjugated goat anti Rabbit IgG(H+L) (Green) served as the secondary antibody. Mitochondria were stained with MitoTracker Red (Red) and DNA was stained with Hoechst 33258 (Blue). White bar, 10 µm.

RNF185 is an evolutionarily conserved gene among vertebrates (Fig. S1). Based on SMART prediction (http://smart.embl-heidelberg.de/), human RNF185 is a 21 kDa protein with a C3HC4 RING domain and two transmembrane (TM) domains, named TM1 and TM2 respectively (Fig. 1A and Fig.S1). In order to define the effect of the RING domain and two TM domains of RNF185 on its subcellular localization, we generated four RNF185 mutants that were fused to GFP (green fluorescent protein) (Fig. 1A). We first transfected the N-terminally GFP-tagged RNF185 (GFP-RNF185-WT) in HeLa cells and stained the cells with organelle-specific fluorescent red dye. The GFP-RNF185 had a clear overlap with MitoTracker Red in the cytoplasmic region (Fig. 1B). While both wild type RNF185 (RNF185-WT) and RING domain mutated RNF185 (RNF185-RM, detailed information on RING mutation is presented in Fig. S2) colocalized well with Mitotracker Red, RNF185 mutant with TM domains deleted (RNF185-TM) was completely mislocalized and distributed evenly in cell nuclei and cytosol. Both the mutation of TM1 (RNF185-TM1, detailed information on TM1 mutation is presented in Fig. S2) and the deletion of TM2 (RNF185-TM2) led to partial mislocalization of RNF185. However, TM2 seemed to be more important for RNF185's correct localization, because more mislocalized GFP-RNF185-TM2 proteins were observed compared with GFP-RNF185-TM1 proteins. Our results revealed that the TM domains play an important role in determining subcellular localization for RNF185.

**Figure 2:**
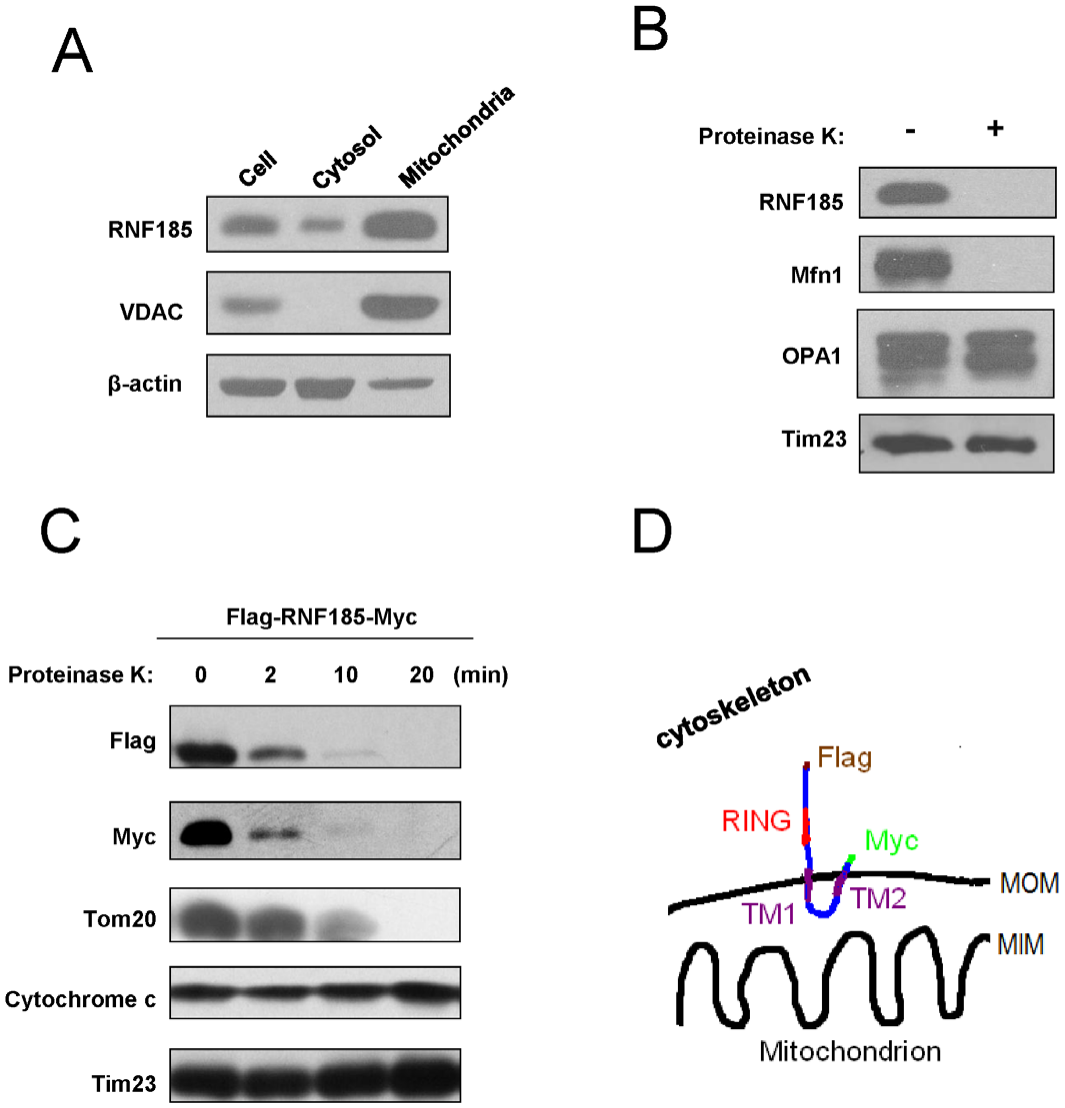
RNF185 localizes at mitochondrial outer membrane with the RING domain exposed to the cytosol. (**A**) 293 cells were subfractionated by differential centrifugation to get mitochondrial fraction (Heavy Membrane, HM) and cytosolic/Light Membrane(LM) fractions. Equal protein amounts (40 µg) of the whole cell extract (cell), cytosolic and LM fraction (cytosol), and HM fraction (Mitochondira) were blotted with anti-RNF185 pAb to detect endogenous RNF185. The mitochondrial protein VDAC and cytosolic protein β-actin served as positive and negative controls respectively for determining mitochondria localized proteins. (**B**) Mitochondria fraction was treated with or without 20µg/ml proteinase K for 20 min on ice, and samples were collected for western blot analysis to detect endogenous RNF185. Mfn1, OPA1 and Tim23 were used as markers for MOM, MIM and intermembrane space proteins respectively. (**C**) Intact mitochondria isolated from 293 cells expressing Flag-RNF185-Myc were treated with 20 µg/ml proteinase K for the indicated time points, and were subjected to western blot analysis with the indicated antibodies. Tom20, cytochrome c and Tim23 were used as markers for MOM, MIM and intermembrane space proteins respectively. (**D**)Topological structure model for Flag-RNF185-Myc at mitochondrial outer membrane. MOM, mitochondrial outer membrane; MIM, mitochondrial inner membrane.

With the mitochondrial targeting sequence fused to the 5′ end of the DsRed2 vector, the mammalian expression vector pDsRed2-Mito is designed for brighter, more persistent and easier labeling of mitochondria[24], [25], [26]. Confocal microscopy showed that DsRed2-Mito and GFP-RNF185 colocalized well in HeLa cells (Fig. 1C). To determine the cellular distribution of endogenous RNF185, we used affinity purified anti-RNF185 polyclonal antibody in conjunction with MitoTracker Red, which is associated with mitochondrion even after cell fixation and permeabilization. We clearly demonstrated that endogenous RNF185 labeled with Alexa Fluor 488 completely overlapped with MitoTracker Red (Fig. 1D and more images in Fig. S3). Moreover, we also used differential centrifugation to substantiate the mitochondrial localization of RNF185. Consistent with previous observations, endogenous RNF185 was the most abundant in the fraction enriched for mitochondria (Fig. 2A). Thus, both cellular and biochemical analyses demonstrate that RNF185 is a mitochondrial protein.

### RNF185 is a MOM protein with RING domain exposed to the cytosol

Mitochondrion is composed of an outer membrane, an intermembrane space, an inner membrane, and the cristae and matrix, with each compartment carrying out specialized functions. To specify the transmembrane location of RNF185, we further performed a proteinase K susceptibility experiment with purified mitochondria. Intact mitochondria were incubated with or without proteinase K and analyzed by western blot to detect RNF185, the mitochondrial outer membrane protein Mfn1, the intermembrane space protein OPA1 and the mitochondrial inner membrane protein Tim23 (Fig. 2B). Endogenous RNF185 was susceptible to proteinase K digestion, and similar result was obtained for Mfn1, while OPA1 and Tim23 remained resistant to proteinase K digestion because of the protection of mitochondrial membranes. To further characterize the topology of RNF185 on mitochondria, we made a construct with a Flag tag and a Myc tag expressed at the N-terminus and C-terminus of RNF185 protein respectively. Mitochondrial fractions isolated from those Flag-RNF185-Myc-expressing cells were subjected to proteinase K digestion at various time points (Fig. 2C). Both the Flag and Myc tags were readily susceptible to proteinase K digestion, and their signals weakened gradually and disappeared within 20 min of treatment. A similar pattern was observed for the MOM protein Tom20. In contrast, cytochrome c and intermembrane space protein Tim23 remained intact. Taken together, our experimental evidence suggests a model for RNF185's subcellular localization on mitochondria (Fig. 2D). Flag-RNF185-Myc is a MOM protein that crosses the membrane twice, exposing its RING domain and short C- terminus to the cytosol.

### RNF185 regulates autophagy

**Figure 3:**
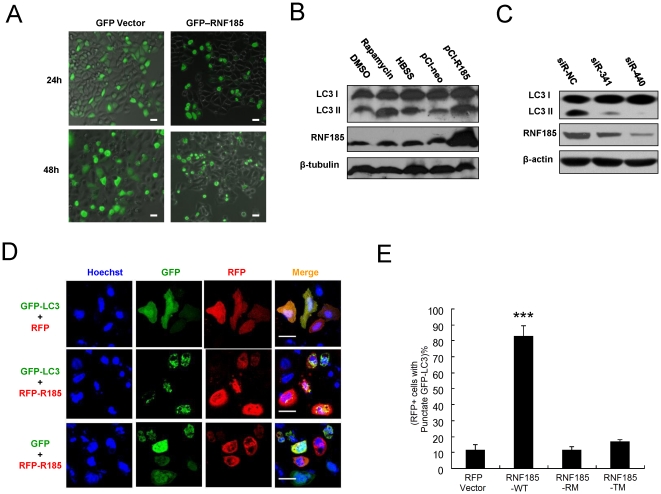
RNF185 regulates autophagy. (**A**) Morphology for HeLa cells that were transiently transfected with GFP tagged RNF185 (GFP-RNF185) or GFP vector alone. Fluorescent microscopic analysis was taken at 24 h and 48 h post transfection. (**B**) Over-expression of RNF185 led to changes in LC3I to LC3II ratio. At 24 h post transfection of empty vector (pCI-neo) and RNF185-expressing vector (pCI-R185), 293T cells were collected for western blot with the indicated antobodies. For positive controls, 293T cells were treated with 10 µM rapamycin for 24 h or maintained in Hank's Buffered Salt Solution (HBSS) for 4 h to induce autophagy. As the vehicle for rapamycin, DMSO served as negative control. (**C**) Knocking down of RNF185 decreased the base level of LC3II. At 36h post transfection of siRNA oligos (siR-341 and siR-440), 293T cells were collected to detect LC3 level by western blot. Bands were detected with a longer exposure time to display the difference on LC3II level. (**D**) Over-expression of RNF185 induced the formation of autophagosome, as detected by the distribution of GFP-LC3 from ubiquitous localization to punctate form. Confocal microscopic analyses were taken at 24 h post co-transfection of the indicated constructs. (**E**) Quantitative analysis of GFP-LC3 vesicular distribution. Experiments were done as in Fig. S6. Data represent mean±SD of 3 independent experiments in which 100–200 RFP+ cells per condition were analyzed. ***, P<0.001. White bar, 20 µm.

HeLa cells with ectopic expression of RNF185 displayed abnormal morphology with globular, shrinking and punctate cell shape (Fig. 3A), which is usually found in dying cells[27]. However, the flow cytometric assay with Annexin V and 7-AAD did not show apoptosis in these cells (Fig. S4A). Moreover, over-expression of RNF185 caused cell cycle arrest and inhibited cell viability (Materials and Methods S1, Fig. S5). A tight relationship between autophagy and cell cycle regulation is revealed by the recently emerging data[28], [29]. To examine whether G1 arrest and abnormal cell shape induced by over-expression of RNF185 are related to autophagy, we performed LC3I to LC3II conversion assay, a typical and simple method to detect signs of autophagy[30]. As shown in Fig. 3B, increased levels of LC3II were observed in cells expressing RNF185 and cells treated with rapamycin or incubated in Hank's Buffered Salt Solution (HBSS). Besides, knocking down RNF185 by siR-341 and siR-440 decreased the base level of LC3II (Fig. 3C). These consistent results suggest that RNF185 is associated with autophagy regulation.

The development of autophagy is frequently assessed by the number and intensity of GFP-LC3 vesicles[31]. To verify whether LC3 is redistributed after over-expression of RNF185, we checked HeLa cells co-transfected with GFP tagged LC3 and RFP (red fluorescent protein) tagged RNF185. The characteristic redistribution of GFP-LC3 was observed, from a diffused cytoplasmic staining in control cells to punctate vesicular structures following over-expressing of RNF185 (Fig. 3D). We also tested the function of RNF185's two mutated forms for induction of autophagy by GFP-LC3 distribution assay (Fig. S6). The percentages of RPF positive cells with obviously punctate GFP-LC3 were greatly increased when wild type RNF185 (RNF185-WT) was expressed, but not for empty vector (RFP Vector), RING domain mutated RNF185 (RNF185-RM) or TM domains deleted RNF185 (RNF185-TM) (Fig. 3E). These results suggested that the induction of punctate GFP-LC3 by over-expressed RNF185 is dependent on its intact RING domain and TM domains.

**Figure 4:**
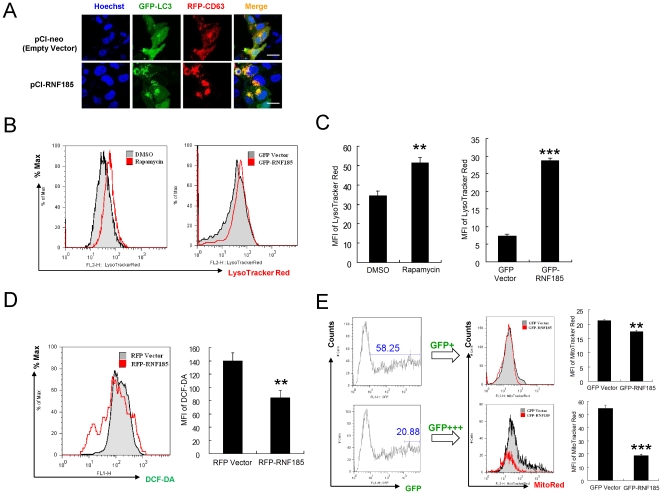
Autophagolysosomes formation and mitochondrial autophagy induced by RNF185 over-expression. (**A**) **–** (**C**) Over-expression of RNF185 promoted the formation of autophagolysosomes. GFP-LC3 and RFP-CD63 colocalized well after the expression of RNF185 (pCI-RNF185), as detected by confocal microscope at 24 h post transfection (**A**). Lysosome accumulation was assayed by LysoTracker Red staining. At 24 h post transfection of empty vector or GFP-RNF185 expressing construct, GFP positive HeLa cells were gated to check the staining of LysoTracker Red by analyzing histogram plot (**B**) and mean fluorescence intensity (MFI) (**C**). (**D**) HeLa cells were transiently transfected with RFP tagged RNF185 (RFP-RNF185) or RFP vector alone, and 24 h later cells were collected for staining by fluorescent probe DCFH-DA to detect the ROS level. The column graph depicts the mean fluorescence intensity of DCF-DA. (**E**) HeLa cells were transiently transfected with GFP tagged RNF185 (GFP-RNF185) or GFP vector alone, and at 24 h post transfection cells were subjected to Mitotracker Red staining. GFP positive cells (GFP+, upper panel) and cells with extremely high GFP intensity (GFP+++, lower panel) were gated to analyze the staining of MitoTracker Red. The column graphs depict the mean fluorescence intensity of MitoTracker Red. Numbers in gates represent percentages of gated cells. n = 3 for each group above. **, P<0.01; ***, P<0.001. White bar, 20 µm.

To promote the degradation of their luminal content, autophagosomes fuse with lysosomes, thus forming the so-called autophagolysosomes[32]. We also detected the accumulation of possible autophagolysosomes using the lysosome membrane marker CD63 (also known as LAMP3, lysosome-associated membrane protein 3) [33]. As shown in Fig. 4A, after over-expression of RNF185, GFP-LC3 accumulated dramatically and overlapped well with RFP tagged CD63. In addition, an increased accumulation of lysosome was observed in HeLa cells that were treated with rapamycin or transfected with expression construct for RNF185 (Fig. 4B and Fig. 4C). A common intracellular stress that effectively leads to induction of autophagy is the formation of ROS. But our results indicated that HeLa cells over-expressed RNF185 had a relative lower level of ROS measured by DCFH-DA staining (Fig. 4D). Mitochondria are the major sources generating ROS, which suggests that the reduced ROS may be caused by the loss of mitochondria mass. Indeed, we observed a dramatic loss of MitoTracker Red staining for the cells with very high level of RNF185 (Fig.4E), implying that the abundance of ectopic expressed RNF185 correlated with the degradation of mitochondria by autophagy.

### RNF185 interacts with BNIP1 and ATG5

**Figure 5:**
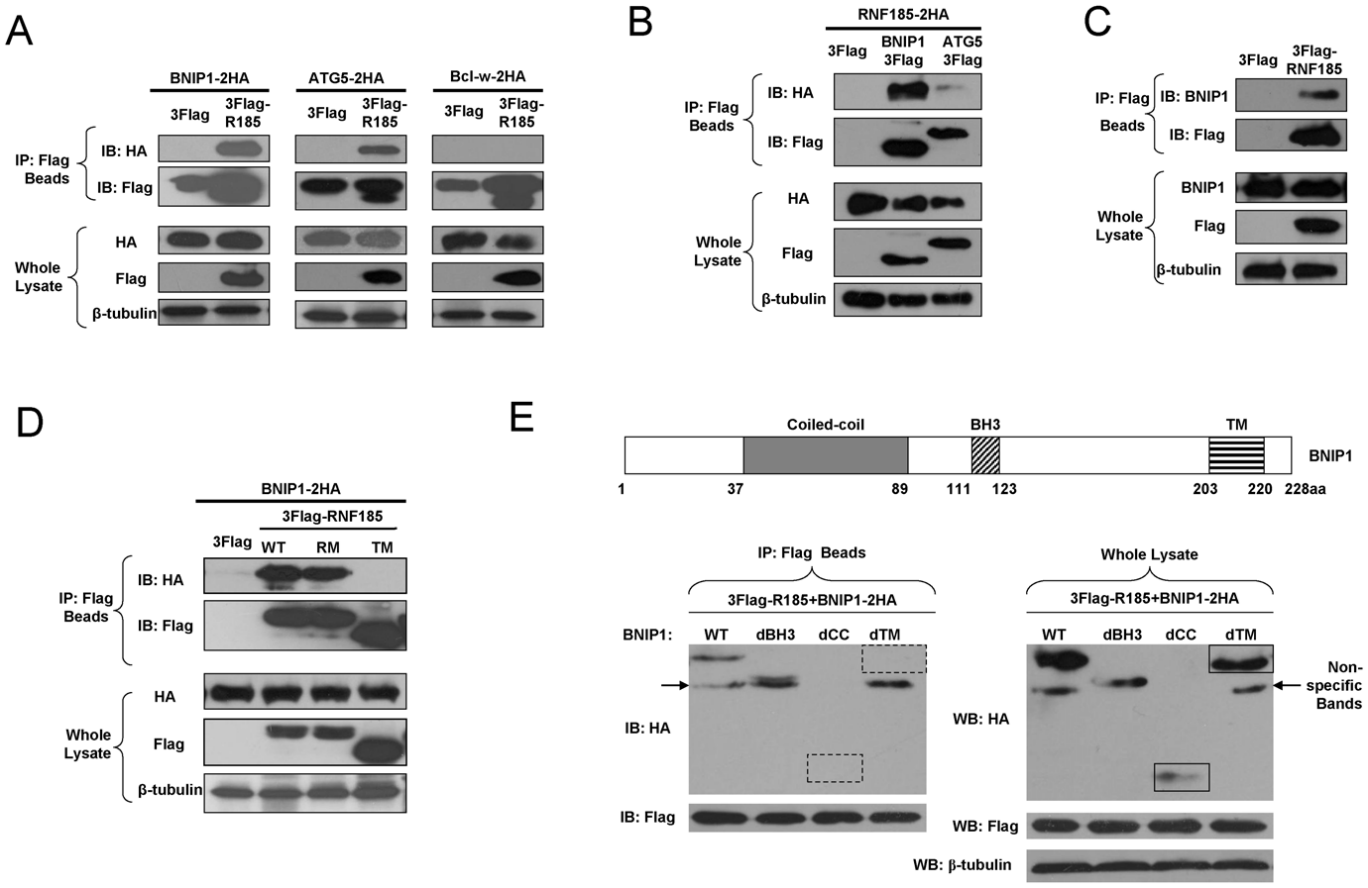
RNF185 interacts with BNIP1 and ATG5. (**A**) RNF185 pulled down BNIP1 and ATG5. 293T cells were co-transfected with expression constructs for 2HA tagged candidate gene and 3Flag tagged RNF185 or empty vector as control. At 24 h post transfection, cell lysates were immunoprecipitated (IP) with anti-Flag conjugated beads and immunoblotted (IB) with anti-Flag and anti-HA antibodies. Bcl-w represented one of the candidate genes that could not interact with RNF185. (**B**) BNIP1 or ATG5 co-immunoprecipitated with RNF185. 3Flag tagged BNIP1 or ATG5 or empty vector and 2HA tagged RNF185 were co-transfected into 293T cells and followed by the same experimental procedures as in (A). HRP-conjugated goat anti mouse/rabbit IgG Fc fragment antibodies were used as secondary antibodies for IB to avoid antibody light chain in (B) to (E). (**C**) Exogenously expressed RNF185 associated with endogenous BNIP1. 293T cells were transfected with 3Flag tagged RNF185 or empty vector, and cell lysates were subjected to IP and IB analysis with the indicated antibodies as in (A). (**D**) The binding of RNF185 to BNIP1 was dependent on its TM domains. RM, RING domain mutated; TM, transmembrane domains deleted. (**E**) RNF185 interacted with the coiled-coil domain of BNIP1. dCC, coiled-coil domain deleted; dBH3, BH3 domain deleted; dTM, transmembrane domain deleted. The arrows indicate non-specific bands. The bands appeared in whole lysates but disappeared after IP are labeled by boxes with solid lines and dotted lines respectively. IP and IB were performed by the same experimental procedures as in (A). β-tubulin was detected as the loading control for all the western blots.

In order to reveal the underlying molecular mechanism for RNF185 induced autophagy, we set out to identify its potential partners. Given the MOM localization of RNF185, we searched for Bcl-2 (B-cell lymphoma 2) family members as they are mitochondria associated proteins and have emerged as regulators of autophagy [34], [35], [36]. Since ATG5 (autophagy related gene 5) is inducibly expressed at mitochondria during autophagy[37], [38], we included ATG5 in our co-transfections consisting of 2HA tagged Bcl-2 family proteins with TM domains (listed in Table S1) and 3Flag tagged RNF185. The results of co-immunoprecipitation clearly demonstrated that BNIP1 (Bcl-2 Nineteen kilodalton Interacting Protein 1) and ATG5 could be pulled down by RNF185 (Fig. 5A). Likewise, RNF185 could also be pulled down by either BNIP1 or ATG5. However, the binding of RNF185 to BNIP1 is apparently stronger than its binding to ATG5 (Fig. 5B). Moreover, exogenously expressed RNF185 was found to associate with endogenous BNIP1 (Fig. 5C). Further investigation indicated that the binding of RNF185 to BNIP1 was dependent on their TM domains(Fig. 5D and Fig. 5E) and the RING domain was not required for RNF185's association with BNIP1 (Fig. 5D). RNF185 seemed to bind the coiled-coil (CC) domain of BNIP1 as 3Flag tagged RNF185 could not pull down dCC mutant of BNIP1 (Fig. 5E).

### The mitochondrial colocalization of BNIP1 and RNF185

**Figure 6:**
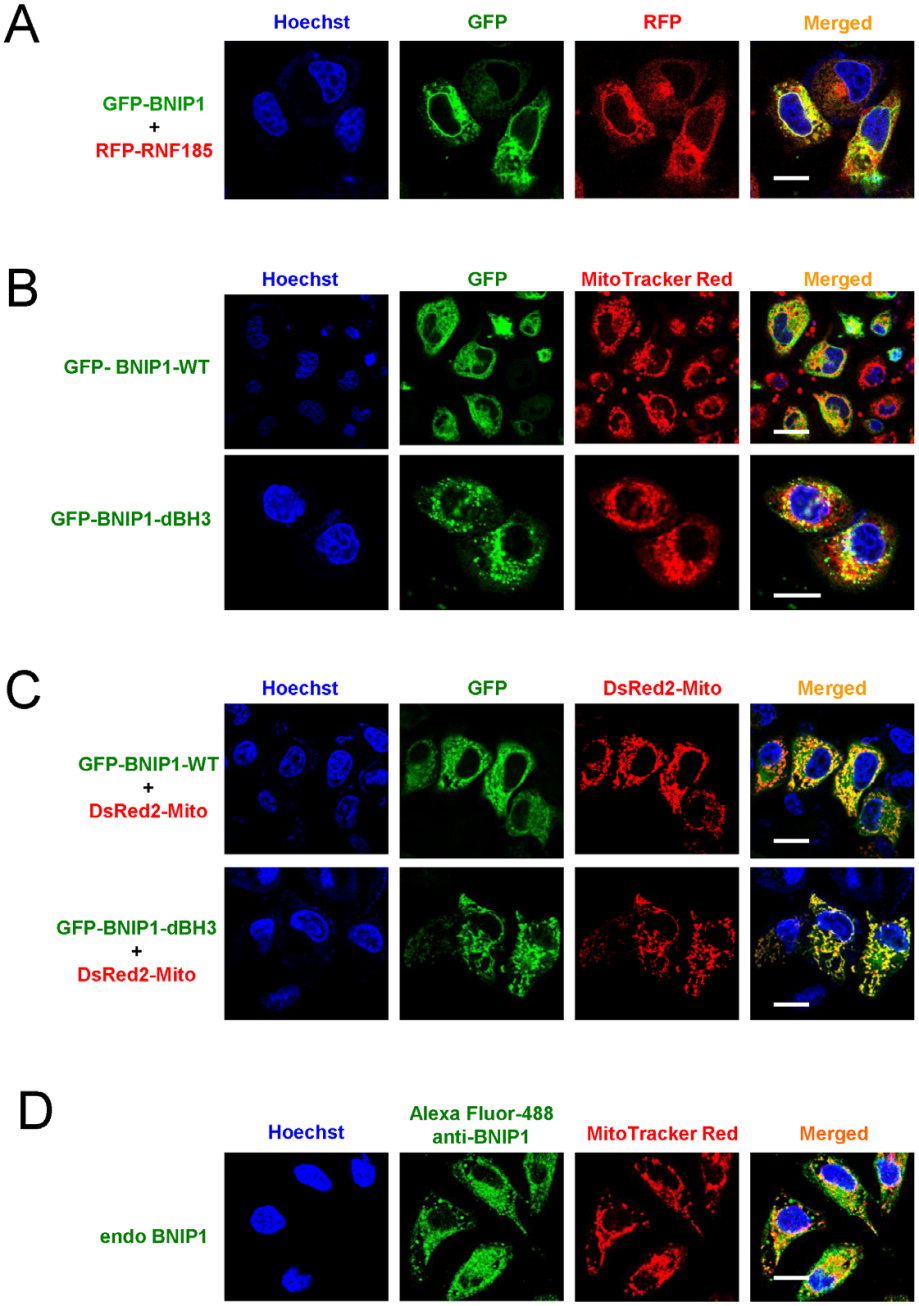
BNIP1 colocalizes with RNF185 at mitochondria. (**A**) Colocalization of RNF185 and BNIP1. Confocal microscopic analysis was taken at 24 h post transfection of GFP tagged BNIP1 and RFP tagged RNF185 in HeLa cells. (**B**) **–** (**D**) BNIP1 localized at mitochondria. Both exogenously expressed wild type BNIP1 (GFP-BNIP1-WT) and mutated BNIP1 with BH3 domain deletion (GFP-BNIP1-dBH3) colocalized well with mitochondria markers Mitotracker Red (**B**) and DsRed2-Mito (**C**). Endogenous BNIP1 overlapped well with Mitotracker Red (**D**). White bar, 20 µm. All the experimental procedures for confocal microscopic analysis were the same as in Figure 1.

Human BNIP1 is a 228-amino acid protein and contains a putative C-terminal TM domain and a BH3 (Bcl-2 homology domain 3) domain (Fig. 5E). Although it has been suggested to localize on the nuclear envelope and/or endoplasmic reticulum (ER)[39], [40], [41], its interaction with RNF185 implied its potential localization at mitochondria. We carried out several experiments to confirm this hypothesis. First, we showed that GFP-BNIP1 and RFP-RNF185 substantially overlapped (Fig. 6A). Second, overlaps between MitoTracker Red and GFP-BNIP1 (Fig. 6B) and between DsRed2-Mito and GFP-BNIP1 (Fig. 6C) were also very strong. It was reported that some pro-apoptotic Bcl-2 family proteins, including BNIP1, translocate to the mitochondria from ER or nuclear membrane to activate downstream signals of apoptosis[41]. The effector BH3 domain in BNIP1 is responsible for its apoptotic function, as BNIP1 mutant lacking BH3 domain could not induce apoptotic cell death[42], [43], [44]. To exclude the possibility that BNIP1 translocates to the mitochondria only after cell undergoes apoptosis, we generated expression construct for mutated BNIP1 with BH3 domain deletion (BNIP1-dBH3). As shown in Fig. 6B and Fig. 6C, GFP-BNIP1-dBH3 colocalized well with both MitoTracker Red and DsRed2-Mito, indicating the intrinsic function of TM domain in BNIP1 for targeting the mitochondria. Noticeably, endogenous BNIP1 was also demonstrated to overlap well with MitoTracker Red (Fig. 6D), which further supports our conclusion of mitochondrial localization for BNIP1.

### RNF185 functions as a ubiquitin E3 ligase, enabling BNIP1-p62 interaction

**Figure 7:**
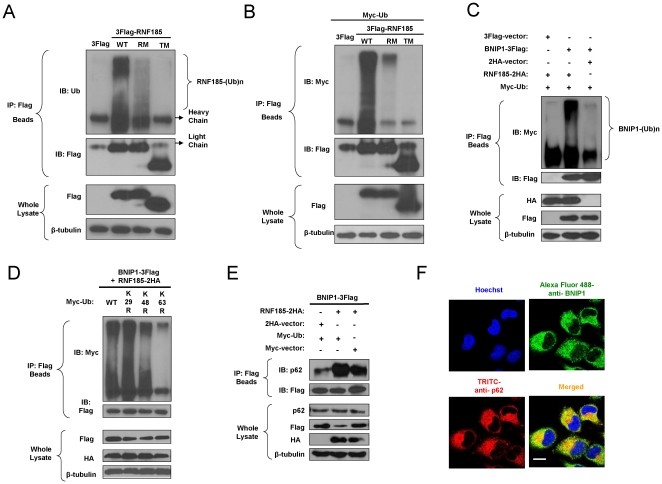
BNIP1 is polyubiquitinated by RNF185 and associates with autophagy receptor p62. (**A**) **–** (**B**) The self-ubiquitination of RNF185 was dependent on its RING domain and TM domains. Empty vector, or plasmid encoding Flag tagged wild type RNF185 or mutant forms of RNF185 was transfected individually (**A**) or cotransfected with Myc tagged ubiquitin (**B**) into 293T cells. Proteins were prepared 24 h after transfection and subjected to IP, followed by IB analysis. Ub, ubiquitin. (**C**) **–** (**D**) RNF185 polyubiquitinated BNIP1 through K63-based ubiquitin linkage. BNIP1 was polyubiquitinated by RNF185 (**C**). 3Flag tagged BNIP1 and 2HA tagged RNF185 were cotransfected with Myc tagged ubiquitin or its variants (K29R, K48R, K63R) into 293T cells, and the polyubiquitination of BNIP1 was detected by IP and IB analysis (**D**). (**E**) BNIP1 associated with p62. 293T cells were cotransfected with the expression constructs for Myc-Ub, 3Flag-BNIP1 and RNF185-2HA or empty vectors as controls. Cell lysates were subjected to IP and IB analysis with the antibodies indicated in the figures. (**F**) Colocalization of endogenous BNIP1 and endogenous p62 in the cytosol. HeLa cells were stained with anti-BNIP1 rabbit polyclonal antibody and anti-p62 mouse monoclonal antibody. Alexa Flour 488-conjugated goat anti-rabbit IgG(H+L) antibody (Green) and TRITC-conjugated goat anti-mouse IgG antibody (Red) were used as secondary antibodies for detection of endogenous BNIP1 and p62 respectively. White bar, 10 µm.

The selfubiquitination of E3 ligase is demonstrable *in vivo* and can be used as a method to assay the ubiquitin E3 ligase activity of RING proteins[45], [46], [47]. First we found that 3Flag tagged RNF185 was intensively polyubiquitinated with endogenous ubiquitin (Fig. 7A) or exogenous ubiquitin (Fig. 7B). The polyubiquitination of RNF185-RM was significantly decreased compared with wild type RNF185, suggesting that the E3 activity of RNF185 is RING domain dependent. Interestingly, the RNF185-TM mutant almost completely lost the activity of self-polyubiquitination, implying that the mitochondrial localization is also critical for RNF185's function as a ubiquitin E3 ligase. To assess whether RNF185 targets BNIP1 ubiquitination *in vivo*, Myc tagged ubiquitin was cotransfected with 2HA tagged RNF185 and 3Flag tagged BNIP1. Ectopically expressed RNF185 caused extensive polyubiquitination of BNIP1 (Fig. 7C). A low level of ubiquitination of BNIP1 was observed in the group without RNF185 transfection, presumably due to endogenous ubiquitin E3 ligases. Using ubiquitin mutants, we observed that BNIP1 was polyubiquitinated to a much lesser degree when the K63 of Myc-ubiquitin was mutated to R63 (Fig. 7D). Therefore, BNIP1 was modified by K63-based polyubiquitin linkage, and this modification was consistent with the self-polyubiquitination pattern of RNF185 (data not shown).

The clearance of protein inclusions by autophagy was promoted by autophagy receptor p62, which preferentially partners with K63-linked polyubiquitin [48], [49], [50], [51]. The association of RNF185 with autophagy regulation and the polyubiquitination of BNIP1 through K63-linkage led us to assess the involvement of p62 in this pathway. Endogenous p62 was detected by western blot after the cotransfection of 3Flag tagged BNIP1, 2HA tagged RNF185 and Myc tagged ubiquitin or vector controls. As shown in Fig. 7E, p62 is co-immunoprecipitated with BNIP1. When both 2HA-RNF185 and Myc-Ub were over-expressed, BNIP1 could recruit much more p62, although endogenous RNF185 and endogenous ubiquitin also contributed to the interaction between p62 and polyubiquitinated BNIP1. In addition, we checked the endogenous localization of BNIP1 and p62 in HeLa cells (Fig. 7F). Alexa Fluor 488 conjugated endogenous BNIP1 and TRITIC conjugated endogenous p62 overlapped well in the cytoplasm, further providing the locational evidence for the recruitment of p62 by BNIP1.

## Discussion

Mitochondria are essential for a variety of cellular functions, including ATP production, lipid biosynthesis, and calcium homeostasis. Recent investigations indicate that certain aspects of mitochondrial functions, including mitochondrial protein quality control and membrane dynamics, are regulated by the ubiquitin-conjugation system[52]. Both MARCH5(RNF153)[53], [54] and MULAN(RNF218)[5], two MOM ubiquitin E3 ligases clearly described so far, were found to be involved in the regulation of mitochondria dynamics. Unlike these MOM E3 ligase, RNF185 does not affect mitochondria fusion and fission (data not shown); whereas RNF185 functions as a specific regulator for autophagy of the mitochondria. The mechanism for the mitochondrial homeostasis by autophagy remained largely unknown. In particular, no MOM E3 ligase has been directly linked to the process. Our data presented herein demonstrate that RNF185 is the MOM E3 ligase responsible for regulation of mitochondrial autophagy. In support of this function, the levels of mouse RNF185 transcript are higher in the tissues and organs (kidney, skeletal muscle, liver and heart) that have higher abundance of mitochondria (unpublished data).

The ubiquitin-conjugation system might be vital for the maintenance of mitochondrial homeostasis and lead to cell demise when dysfunctional[52]. Parkin, an E3 ubiquitin ligase that is mutated in monogenic forms of Parkinson's disease, was recently found to induce selective autophagy of damaged mitochondria. Studies from different laboratories demonstrate that PINK1 is selectively stabilized on impaired mitochondria to activate latent Parkin for mitophagy[55], [56], [57], [58], [59]. Parkin and RNF185 seem to function in different ways. First, distinct from RNF185 which is a resident MOM E3 ligase, endogenous Parkin predominately locates in the cytosol under normal physiological conditions and translocates to mitochondria only after their depolarization. Second, RNF185 can trigger autophagy in HeLa cells, which have little or no endogenous Parkin expression[56], [57]. These facts suggest that RNF185 functions independently of Parkin for mitophagy induction. Parkin induces the specific elimination of damaged mitochondria, while RNF185 seems to play a constitutive role in the modulation of mitochondria homeostasis. However, the autophagy adaptor molecule p62 is involved in both RNF185- and Parkin-mediated clearance of mitochondria by autophagy[56]. It is reported that after translocation to mitochondria, Parkin activates the ubiquitin-proteasome system for widespread degradation of MOM proteins, which is critical for mitophagy[60]. Whether RNF185 can cause the broad ubiquitination of known MOM proteins and how RNF185 functionally relates to Parkin under stress conditions such as mitochondria depolarization, need to be further investigated.

The marked correlation between cell cycle and autophagy has been investigated recently, and the results showed that autophagy is stereotypically induced in the G1 and S phases of the cell cycle [28]. Our findings on G1 arrest (Fig. S5) and autophagy induction (Fig. 3 and Fig. S6) by RNF185 over-expression provide new evidence for the crosstalk between cell cycle regulation and autophagic vacuolization. Cells normally switch between apoptosis and autophagy in a mutually exclusive manner for the same cellular settings[61], we indeed observed that RNF185 had the capacity of inhibiting apoptosis to some extent (Fig. S4). In mammals, increasing data demonstrated that Bcl-2 family proteins play a dual role in the control of apoptosis and autophagy. Recent investigation indicates that cellular anti-apoptotic proteins such as Bcl-2, Bcl-xl, Bcl-w can inhibit autophagy[62], [63], [64], while pro-apoptotic BH3-only proteins from the Bcl-2 family such as BNIP3, Bad, Bik can induce autophagy[65], [66], [67], [68], via their differential interaction with Beclin 1. Here we identified BNIP1, another pro-apoptotic BH3-only member of Bcl-2 family proteins, as a critical player in autophagy induction. BNIP1 regulates autophagy mainly through recruiting autophagy receptor p62 to mitochondria, rather than competitively disrupting the interaction between Beclin1 and Bcl-2/Bcl-w/Bcl-xl, which implies another possible way of crosstalk between apoptosis and autophagy.

The term “mitophagy” was created to describe the selective removal of mitochondria by autophagy, but the signals and specificity in targeting mitochondria to the autophagy pathway remained poorly understood. The mitochondria-localized proteins BNIP3 and NIX have been implicated in the removal of mitochondria during hypoxia-induced autophagic responses[67], [68]. Recently, a novel mitochondrial protein, Atg32, was characterized as a selective autophagy receptor for autophagic degradation of stressed mitochondria in yeast[69], [70]. NIX was proposed as a counterpart of Atg32 in higher organisms because it binds LC3/GABARAP and mediates mitochondrial clearance in murine reticulocytes[71], [72]. However, the proteins reported above do not account for several important events, such as ubiquitination of mitochondrial proteins and interactions with lysosomal components, which might mediate the full incorporation of mitochondria into autophagosome. Our findings on the function of mitochondrial ubiquitin E3 ligase RNF185 might reveal a novel mechanism for modulating mitochondria homeostasis through autophagy.

**Figure 8:**
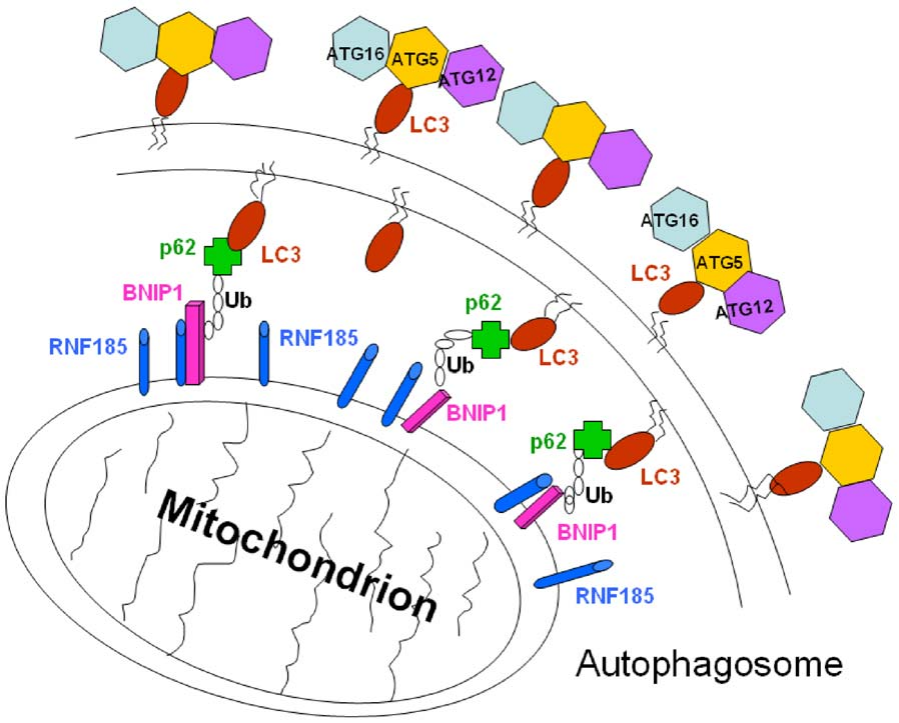
A proposed model for RNF185 mediated autophagy regulation. Mitochondrial outer membrane E3 ubiquitin ligase RNF185 interacts with its substrate BNIP1, which also localizes at mitochondria. BNIP1 is polyubiquitinated by RNF185 through K63-based ubiquitin linkage, and recruits autophagy receptor p62, which can interact with both LC3 and ubiquitins. The accumulation of p62 and LC3 promotes the formation of autophagosome. Ub, ubiquitin; ATG12-ATG5-ATG16, the protein complex required for the expansion of the autophagosomal membrane.

We proposed a model for RNF185 mediated selective degradation of mitochondria by autophagy (Fig. 8). Both RNF185 and BNIP1 localize at mitochondria, and BNIP1 is modified with K63-based polyubiquitin linkage by RNF185. The polyubiquitinated BNIP1 recruits autophagy receptor p62, which binds both ubiquitins and LC3/GABARAP. The accumulation of LC3/GABARAP proteins anchored in the double membrane of the forming autophagosome promotes the degradation of mitochondria in lysosomes. The over-expression of RNF185 was associated with GFP-LC3 distribution and its overlap with RFP-CD63, as well as the higher level of LysoTracker Red staining (Fig. 4). All of these facts imply the formation of autophagolysosome. It is identified recently that the outer membrane of mitochondria is a new source of autophagosomal membranes during starvation, and the mitochondrial outer membrane marker is present on membrane of autophagosomes[73], [74]. Importantly, Atg5, which is essential for the recruitment of LC3 and the expansion of autophagosomes, also localized with LC3 to mitochondria's outer membranes[75]. Our finding on the interaction between RNF185 and Atg5 (Fig. 5) also suggests a possible involvement of RNF185 in the regulation of autophagy by promoting the autophagosome biogenesis from mitochondrial outer membrane. In addition, we also observed that RFP-RNF185 had some overlaps with GFP-LC3 (Fig. 3D and Fig. S6), and GFP-RNF185 partially colocalized with LysoTracker Red and RFP-CD63 (data not shown). These findings further support our conclusion that RNF185 is a positive regulator for the formation of autophagolysosome.

The induction of autophagy, however, did not affect the RNF185 mRNA transcript (data not shown) and protein (Fig. 3B) levels. This probably implies that RNF185 employs a different ubiquitination related pathway for selective autophagy regulation rather than the classical mTOR (mammalian target of rapamycin) pathway activated by rapamycin or deprivation of nutrient[76]. In addition, the evolutionary conservation in vertebrates (Fig. S1) and wide distribution in human cells and mouse tissues (data not shown) imply RNF185 may play critical roles in the fundamental biological processes, of which modulating mitochondria homeostasis through autophagy is an essential one. Targeted degradation of mitochondria by autophagy is an important catabolic process that can be utilized for new therapeutic approaches in treatment of cancer and mitochondria related diseases[77], [78]. We propose that RNF185 induced ubiquitination on mitochondrial membrane protein BNIP1 provides a signal that leads to the autophagosome formation. The therapeutic approaches targeting this signal will hold promise of an exciting opportunity to modulate levels of selective mitochondrial autophagy in different pathological conditions.

## Materials and Methods

### Cell culture and transfection

HeLa, 293 and 293T cells were originally obtained from ATCC (American Type Culture Collection). Cells were grown in Dulbecco's Modified Eagle's Medium (DMEM, Gibco) supplemented with 10% heat-inactivated fetal bovine serum (FSS500, Hyclone), penicillin (100 U/ml) and streptomycin (100 µg/ml). 293 cells expressing Flag-RNF185-Myc were grown in complete DMEM supplemented with G418 (200 µg/ml, Sigma). All cells were maintained at 37°C in a humidified atmosphere with 5% CO2. Cells were transfected with the indicated plasmids or siRNA oligos using Lipofectamine 2000 (Invitrogen) according to the manufacture's protocol. The sequences for all the siRNA oligos are as following: siR-NC, sense 5′-UUCUCCGAACGUGUCACGUTT-3′, anti-sense 5′-ACGUGACACGUUCGGAGAATT-3′;siR-341, sense 5′-GGCCAGAGCCGGAGAAUAGTT-3′, anti-sense 5′-CUAUUCUCCGGCUCUGGCCTT-3′; siR-440, sense 5′-GCCACAGCAUUUAAUAUAATT-3′, anti-sense 5′-UUAUAUUAAAUGCUGUGGCAA-3′.

### Antibodies and chemicals

The recombinant RNF185-132 protein (corresponding to amino acids 1-132 of human RNF185, with transmembrane domains deleted) purified from *E.coli* was used for rabbit immunization. Highly specific polyclonal antibody (pAb) against RNF185 was obtained by antigen affinity chromatography via CNBr-activated Sepharose 4B (GE Healthcare) according to the manufacturer's instructions. Other primary antibodies used in this study were: Flag, β-tubulin, β-actin (Sigma); Myc, Mfn1, Ubiquitin, VDAC, GFP (Santa Cruz); OPA1, Tim23, Tom20, Cytochrome c (BD Pharmingen); BNIP1 (ProteinTech Group); p62 (BD Transduction Laboratories). Secondary antibodies used were: Alexa Fluor 488 goat anti-rabbit IgG(H+L) (Invitrogen), TRITC goat anti-mouse IgG(H+L) (Zymed Laboratories), horseradish peroxidase (HRP) conjugated goat anti-rabbit/mouse IgG(H+L) (Sigma), HRP conjugated goat anti-mouse Fc fragment (Thermo Scientific) and HRP conjugated goat anti-rabbit Fc fragment (Jackson ImmunoResearch Laboratories). All other chemicals and reagents were from Sigma-Aldrich, Inc.

### Construction of plasmids

The full length cDNAs for human RNF185 (GenBank accession No. NM_152267), CD63 (NM_001780), and ubiquitin (NM_018955) were obtained from cDNA library of HeLa cells by RT-PCR. Other cDNAs were from ProteinTech Group, Inc (Chicago, IL, USA). The truncated RNF185**–**132 construct was subcloned into a vector named pET41d (a pET41a variant with the GST coding sequences deleted) between the EcoRI and SalI sites for protein expression and purification. Vectors used to generate tagged wild type or mutated RNF185/BNIP1 were p3XFlag-CMV10 (sigma) and pCI-neo (Promega). RNF185 was subcloned into pcDNA4/TO/myc-HisTMB (Invitrogen) via HindIII and EcoRV sites to get RNF185-Myc expressing plasmid, which was double digested by enzyme HindIII and EcoRI. And the digested fragment was ligated into pFlag-CMV4 (Sigma) to get the plasmid expressing Flag-RNF185-Myc. Myc tagged wild type and mutated ubiquitins (K29R, K48R and K63R) were ligated to pCI-neo vector via EcoRI and XbaI sites. All constructs were verified by sequencing.

### Immunocytochemistry, confocal microscopy and flow cytometry

HeLa cells grown on 35 mm glass bottom dishes were washed by phosphate-buffered saline (PBS) for 3 times and fixed with 4% paraformaldehyde in PBS for 15 min at room temperature (RT). After washing with PBS, cells were permeabilized with 0.1% Triton X-100 for 5 min on ice, washed again with PBS and blocked with 5% goat serum in PBS for 1 h at RT. Cells were then incubated with first antibodies in a humidity chamber at 4°C overnight. The next day cells were washed for 3 times by PBS before incubation with secondary antibodies diluted in PBS for 20 min at RT and washed again for 3 times. Living cells were incubated with 100 nM MitoTracker Red (CMXRos, Invitrogen) or 50 nM LysoTracker Red (Invitrogen) in DMEM for 30 min at 37°C to stain mitochondria and lysosomes respectively. For quantification of autophagy, HeLa cells were blindly classified as autophagy negative cells (that present a predominant diffuse GFP-LC3) or autophagy positive cells (cells with a punctate GFP-LC3 pattern) at 24 h post transfection. Immunofluorescence data were obtained using Olympus Fluoview 500 laser scanning confocal microscope and analyzed by Image J software (National Institutes of Health, USA). Cytometric analyses were performed using a flow cytometer (FACS Calibur, Becton Dickinson) and FlowJo software (Tree Star).

### Preparation of mitochondrial and cytosolic fractions

293 cells were harvested from 100 mm dishes and washed with mitochondrial isolation buffer (10 mM HEPES-KOH, pH7.2, containing 1.5 mM MgCl2, 1 mM EDTA, 1 mM EGTA, 0.21 M sucrose, 70 mM mannitol and protease inhibitors). Approximate 1X107 cells resuspended in 1 ml mitochondrial isolation buffer were kept on ice for 30**–**60 min with frequent tapping. The cellular suspension was homogenized with a glass Dounce homogenizer with 50 times up and down passes of the pestle. The homogenate was centrifuged at 1000 g for 10 min at 4°C to remove the nuclei and intact cells, and the supernatant was centrifuged at 10 000 g for 15 min at 4°C. The resulting supernatant (cytosolic fraction) was removed while the pellet (mitochondrial fraction) was collected for downstream applications.

### Immunoprecipitation and Western blot

Cells were lysed for 30 min on ice in lysis buffer (50 mM Tris-HCl, pH7.5, 150 mM NaCl, 1 mM EDTA, 1% Triton X-100, 10% glycerol and protease inhibitors). The lysates were centrifuged at 15 000 g for 15 min at 4°C and the supernatant was collected. Antibody was added to the supernatant and incubated for 3 h with rotation at 4°C. The immunecomplex was then precipitated with protein A/G-sepharose beads (Pierce) according to manufacture's instructions. The precipitated samples were boiled and separated by 10%**–**15% SDS-PAGE, then transferred to PVDF membrane (Millipore). The membrane was probed with the indicated primary antibody over night at 4°C after blocking with 10% skim milk in TBS-T (Tris-Buffered Saline supplemented with 0.1% Tween 20), and followed by incubation with an appropriate secondary HRP-conjugated antibody for 1 h at RT after washing with TBS-T. Bands were detected using enhanced chemiluminescence kit (ECLTM, GE Healthcare) as per manufacturer's instructions.

### Statistical analysis

All the data are presented as mean ± SD. The two-tailed, paired student's *t* tests were used for comparison between two experimental groups. Statistical significance was determined as P<0.05.

## Supporting Information

Figure S1 **Gene architecture of RNF185 and conservation analysis.** RNF185 proteins are evolutionarily conserved among vertebrates. The orthologs from chimpanzee(XP_515084), pig(XP_001925859), mouse(NP_663330, with the first 36 amino acids deleted), rat(NP_001019442), dog(XP_852634), cattle(NP_001077172), chicken(NP_001007841), hoptoad(NP_001088405) and zebrafish(NP_998202) were compared with human RNF185(NP_689480) by alignment of the amino acids sequences. The red asterisks indicate the conserved residues in a canonical RING domain. Underlined sequences represent regions predicted to be TM domains.
(TIF)

Figure S2 **The depiction of mutation for RING domain and TM1 domain.** The Zn2+ binding C3HC4 RING domain of RNF185 was completely destroyed by replacing the central three cysteine (C) residues and one histidine (H) residue with alanine (A) and tryptophan (W) residues respectively. The TM1 domain (133aa to 154aa of RNF185) was mutated by replacing the amino acids with hydrophilic and polar residues Arg, Asp and Glu for every five residues. The demolishment of this hydrophobic region was confirmed by the TMPred Server (http://www.ch.embnet.org/software/TMPRED_form.html).
(TIF)

Figure S3 **More images for the mitochondrial localization of endogenous RNF185.** HeLa cells were analyzed by confocal microscope after staining with MitoTracker Red and affinity chromatography purified highly specific polyclonal antibody raised against RNF185. Alexa fluor 488 goat anti Rabbit IgG(H+L) (Green) served as the secondary antibody. White bar, 10 µm.
(TIF)

Figure S4 **RNF185 negatively regulates apoptosis.** (**A**)Over-expression of RNF185 did not induce apoptosis. HeLa cells were transiently transfected with GFP tagged RNF185 (GFP-RNF185) or GFP vector alone. GFP+ cells were gated to analyze the induction of apoptosis by PE-Annexin V and 7-AAD staining at 36h post transfection. Numbers in quadrants represent frequencies. (**B**)**–**(**D**)Over-expression of RNF185 inhibited etoposide induced cell apoptosis. At 20 h after transfection, HeLa cells were treated with 300 µM etoposide for an additional 4 h before being collected for apoptosis analysis. GFP+ cells were gated to analyze the staining of PE-Annexin V and 7-AAD (**B**). The histogram plots of 7-AAD staining (**C**) and PE-Annexin V staining (**D**) for GFP+ cells are displayed. Numbers in quadrants represent frequencies. (**E**) **–** (**F**) Knocking down of RNF185 increased the sensitivity to apoptosis induction. HeLa cells were transiently transfected with RNF185 specific siRNAs (siR-341 and siR-440), or with a non-specific control siRNA (siR-NC), and 24 h later the cells were treated with 20 µM etoposide for an additional 24 h before being collected for apoptosis analysis. The representative histograms depicting PE-Annexin V staining are displayed (**E**), and numbers in gates represent percentages of PE-Annexin V positive cells. Knocking down of RNF185 significantly increased the percentages of PE-Annexin V positive cells (**F**). n = 4 for each group. **, P<0.01; ***, P<0.001.
(TIF)

Figure S5 **RNF185 is involved in the control of cell cycle and cell growth.** (**A**) **–** (**B**)HeLa cells were transfected with siRNAs (siR-341 and siR-440) targeting two different sequences of RNF185 mRNA, or with a negative control siRNA (siR-NC). At 36 h post transfection, knocking down efficiency was determined by conventional RT-PCR (**A**) and western blot (**B**). **(C)**Knocking down of endogenous RNF185 led to decreased G1 phase population and increased S phase population. At 36 h after transfection with the indicated siRNA oligos, HeLa cells were harvested for cell cycle assay. (**D**) Ectopic expression of RNF185 caused G1 arrest. GFP alone or GFP fused with wild type RNF185 and its mutants were individually over-expressed in HeLa cells, and the GFP+ fractions were gated for cell cycle analysis at 24 h post transient transfection. WT, wild type; RM, RING domain mutated; TM, both TM1 and TM2 were deleted. (**E**) Control 293 cells and RNF185-Myc inducible 293 cells were incubated in culture medium with or without 1 µg/ml tetracycline (Tet) for 24 h. Cells were harvested for western blot analysis using the indicated antibodies. β-tubulin served as internal control to assure equal loading. (**F**) Over-expression of RNF185 inhibited cellular proliferation. MTT proliferation assay was performed in control 293 cells and RNF185-Myc Tet-On inducible 293 cells with or without treatment by tetracycline. Data represent one of five independent experimental results. n = 6 for each group. *, P<0.05; **, P<0.01; NS, not significant.
(TIF)

Figure S6 **The induction of punctate GFP-LC3 by ectopic expression of RNF185 depends on its RING domain and TM domains.** Confocal microscopic analyses were taken at 24 h post the co-transfection of the indicated constructs. WT, wild type; RM, RING domain mutated; TM, transmembrane domains deleted. White bar, 10 µm.
(TIF)

Table S1 **List of Bcl-2 family proteins with transmembrane domains.**
(DOC)

Materials and Methods S1 **Analysis of cell cycle and Cell proliferation assay.**
(TIF)
