# Supplementary figures and images for: RNF185, a Novel Mitochondrial Ubiquitin E3 Ligase, Regulates Autophagy through Interaction with BNIP1
Source: PLoS One. 2011 Sep 9;6(9):e24367. doi: 10.1371/journal.pone.0024367 (PMC3170314; doi:10.1371/journal.pone.0024367)

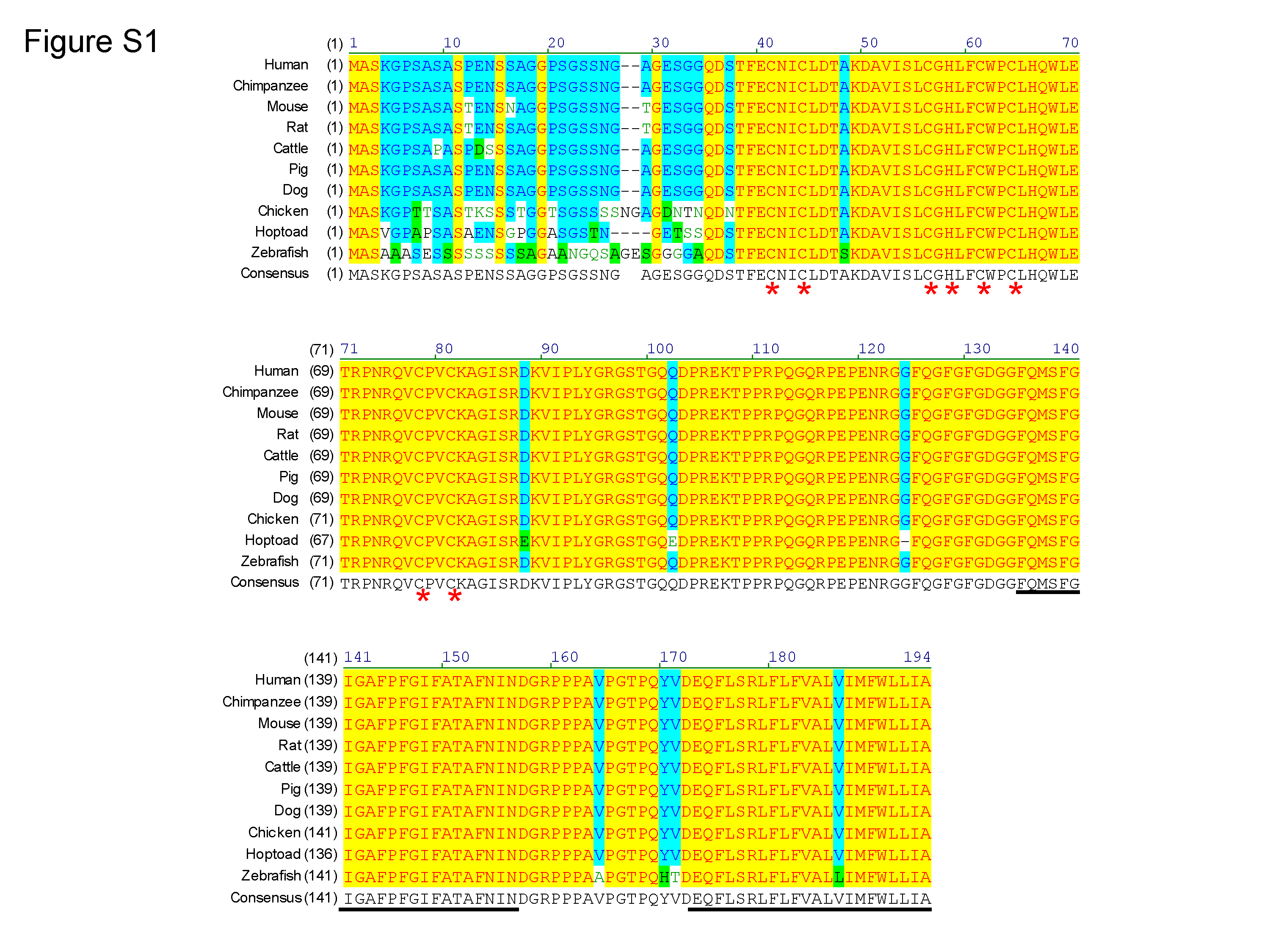

Supplement: Figure S1 — Gene architecture of RNF185 and conservation analysis. RNF185 proteins are evolutionarily conserved among vertebrates. The orthologs from chimpanzee(XP_515084), pig(XP_001925859), mouse(NP_663330, with the first 36 amino acids deleted), rat(NP_001019442), dog(XP_852634), cattle(NP_001077172), chicken(NP_001007841), hoptoad(NP_001088405) and zebrafish(NP_998202) were compared with human RNF185(NP_689480) by alignment of the amino acids sequences. The red asterisks indicate the conserved residues in a canonical RING domain. Underlined sequences represent regions predicted to be TM domains. (TIF) [file pone.0024367.s001.tif]

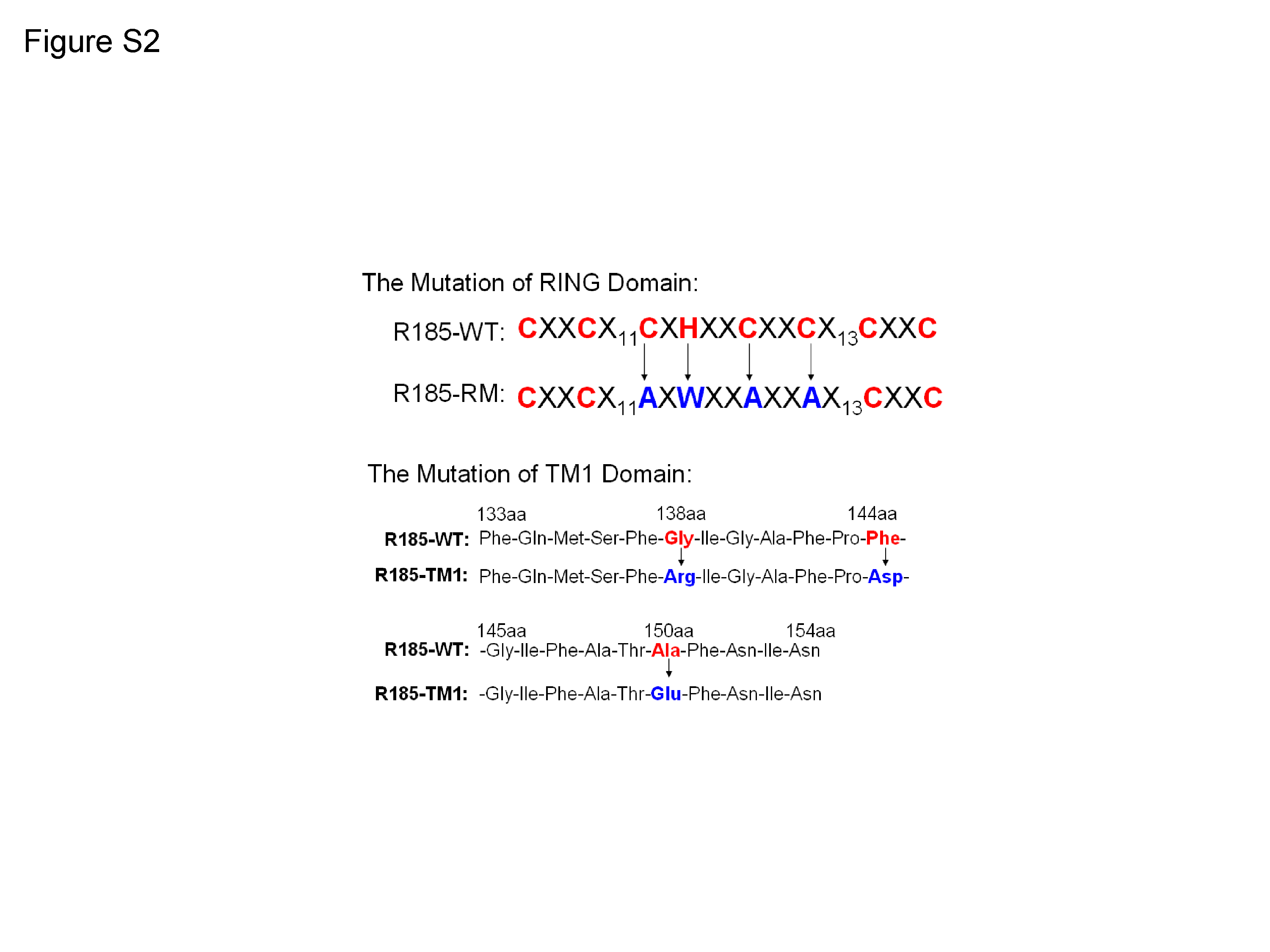

Supplement: Figure S2 — The depiction of mutation for RING domain and TM1 domain. The Zn2+ binding C3HC4 RING domain of RNF185 was completely destroyed by replacing the central three cysteine (C) residues and one histidine (H) residue with alanine (A) and tryptophan (W) residues respectively. The TM1 domain (133aa to 154aa of RNF185) was mutated by replacing the amino acids with hydrophilic and polar residues Arg, Asp and Glu for every five residues. The demolishment of this hydrophobic region was confirmed by the TMPred Server (http://www.ch.embnet.org/software/TMPRED_form.html). (TIF) [file pone.0024367.s002.tif]

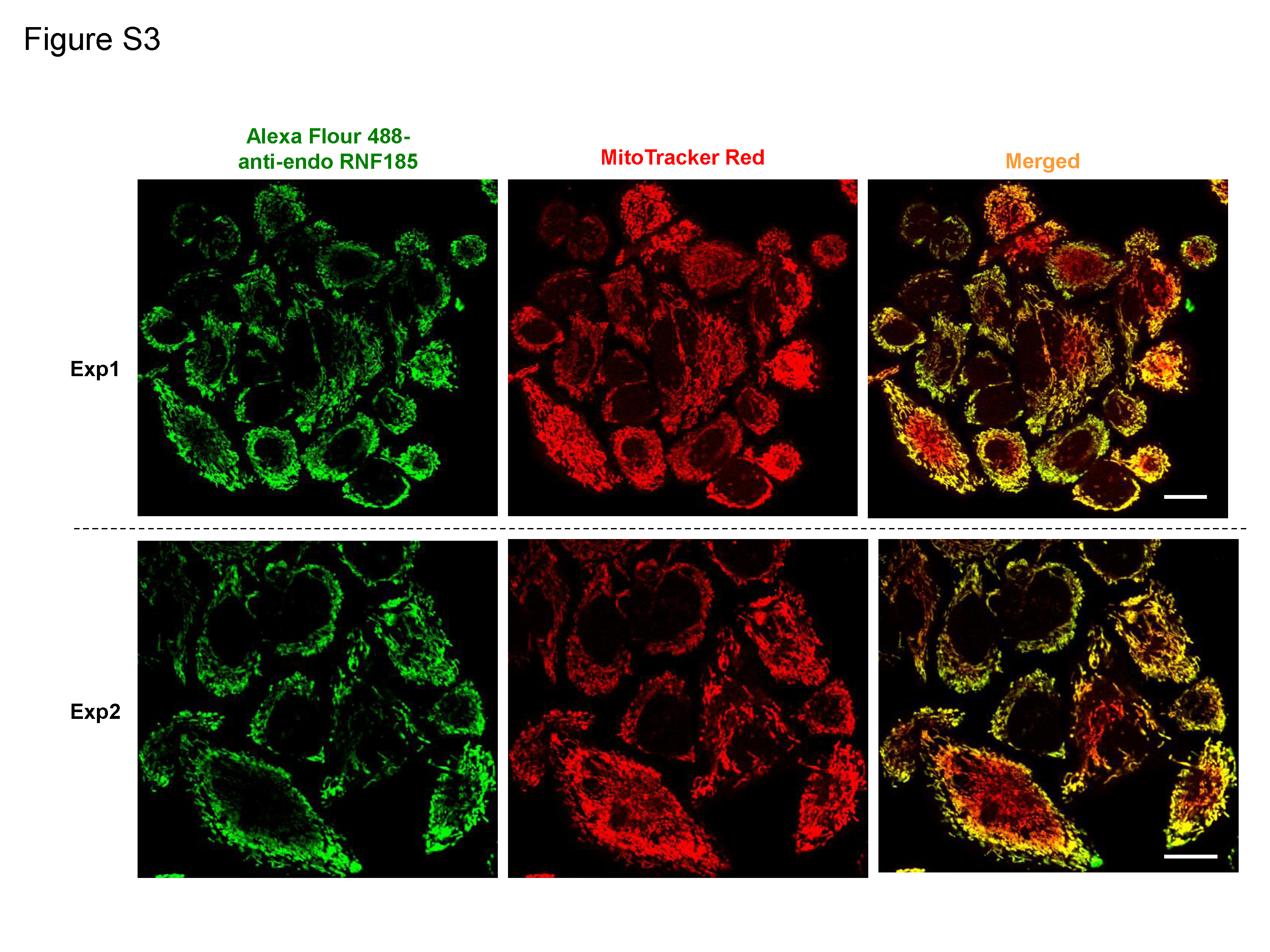

Supplement: Figure S3 — More images for the mitochondrial localization of endogenous RNF185. HeLa cells were analyzed by confocal microscope after staining with MitoTracker Red and affinity chromatography purified highly specific polyclonal antibody raised against RNF185. Alexa fluor 488 goat anti Rabbit IgG(H+L) (Green) served as the secondary antibody. White bar, 10 µm. (TIF) [file pone.0024367.s003.tif]

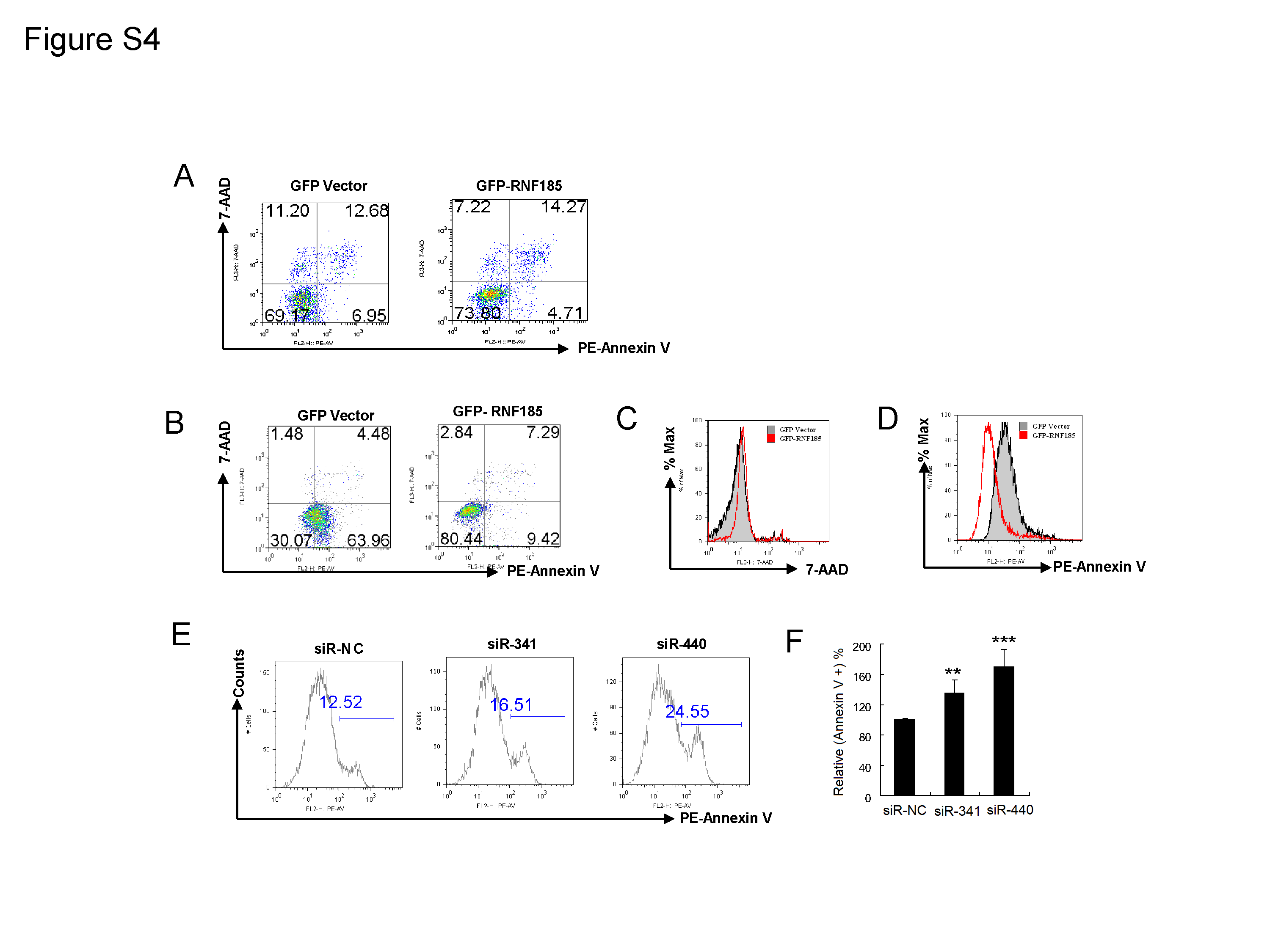

Supplement: Figure S4 — RNF185 negatively regulates apoptosis. (A)Over-expression of RNF185 did not induce apoptosis. HeLa cells were transiently transfected with GFP tagged RNF185 (GFP-RNF185) or GFP vector alone. GFP+ cells were gated to analyze the induction of apoptosis by PE-Annexin V and 7-AAD staining at 36h post transfection. Numbers in quadrants represent frequencies. (B)–(D)Over-expression of RNF185 inhibited etoposide induced cell apoptosis. At 20 h after transfection, HeLa cells were treated with 300 µM etoposide for an additional 4 h before being collected for apoptosis analysis. GFP+ cells were gated to analyze the staining of PE-Annexin V and 7-AAD (B). The histogram plots of 7-AAD staining (C) and PE-Annexin V staining (D) for GFP+ cells are displayed. Numbers in quadrants represent frequencies. (E) – (F) Knocking down of RNF185 increased the sensitivity to apoptosis induction. HeLa cells were transiently transfected with RNF185 specific siRNAs (siR-341 and siR-440), or with a non-specific control siRNA (siR-NC), and 24 h later the cells were treated with 20 µM etoposide for an additional 24 h before being collected for apoptosis analysis. The representative histograms depicting PE-Annexin V staining are displayed (E), and numbers in gates represent percentages of PE-Annexin V positive cells. Knocking down of RNF185 significantly increased the percentages of PE-Annexin V positive cells (F). n = 4 for each group. **, P<0.01; ***, P<0.001. (TIF) [file pone.0024367.s004.tif]

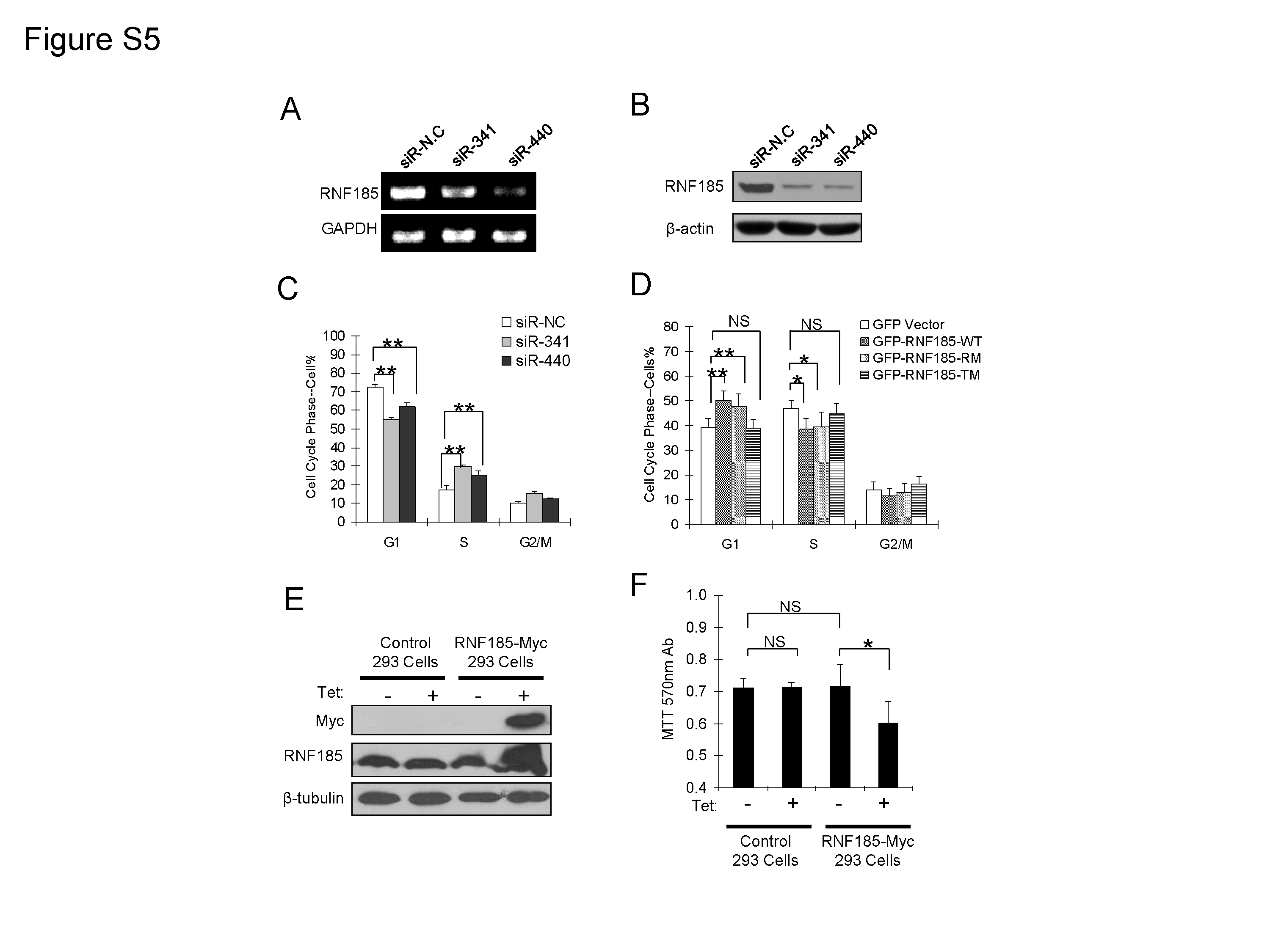

Supplement: Figure S5 — RNF185 is involved in the control of cell cycle and cell growth. (A) – (B)HeLa cells were transfected with siRNAs (siR-341 and siR-440) targeting two different sequences of RNF185 mRNA, or with a negative control siRNA (siR-NC). At 36 h post transfection, knocking down efficiency was determined by conventional RT-PCR (A) and western blot (B). (C)Knocking down of endogenous RNF185 led to decreased G1 phase population and increased S phase population. At 36 h after transfection with the indicated siRNA oligos, HeLa cells were harvested for cell cycle assay. (D) Ectopic expression of RNF185 caused G1 arrest. GFP alone or GFP fused with wild type RNF185 and its mutants were individually over-expressed in HeLa cells, and the GFP+ fractions were gated for cell cycle analysis at 24 h post transient transfection. WT, wild type; RM, RING domain mutated; TM, both TM1 and TM2 were deleted. (E) Control 293 cells and RNF185-Myc inducible 293 cells were incubated in culture medium with or without 1 µg/ml tetracycline (Tet) for 24 h. Cells were harvested for western blot analysis using the indicated antibodies. β-tubulin served as internal control to assure equal loading. (F) Over-expression of RNF185 inhibited cellular proliferation. MTT proliferation assay was performed in control 293 cells and RNF185-Myc Tet-On inducible 293 cells with or without treatment by tetracycline. Data represent one of five independent experimental results. n = 6 for each group. *, P<0.05; **, P<0.01; NS, not significant. (TIF) [file pone.0024367.s005.tif]

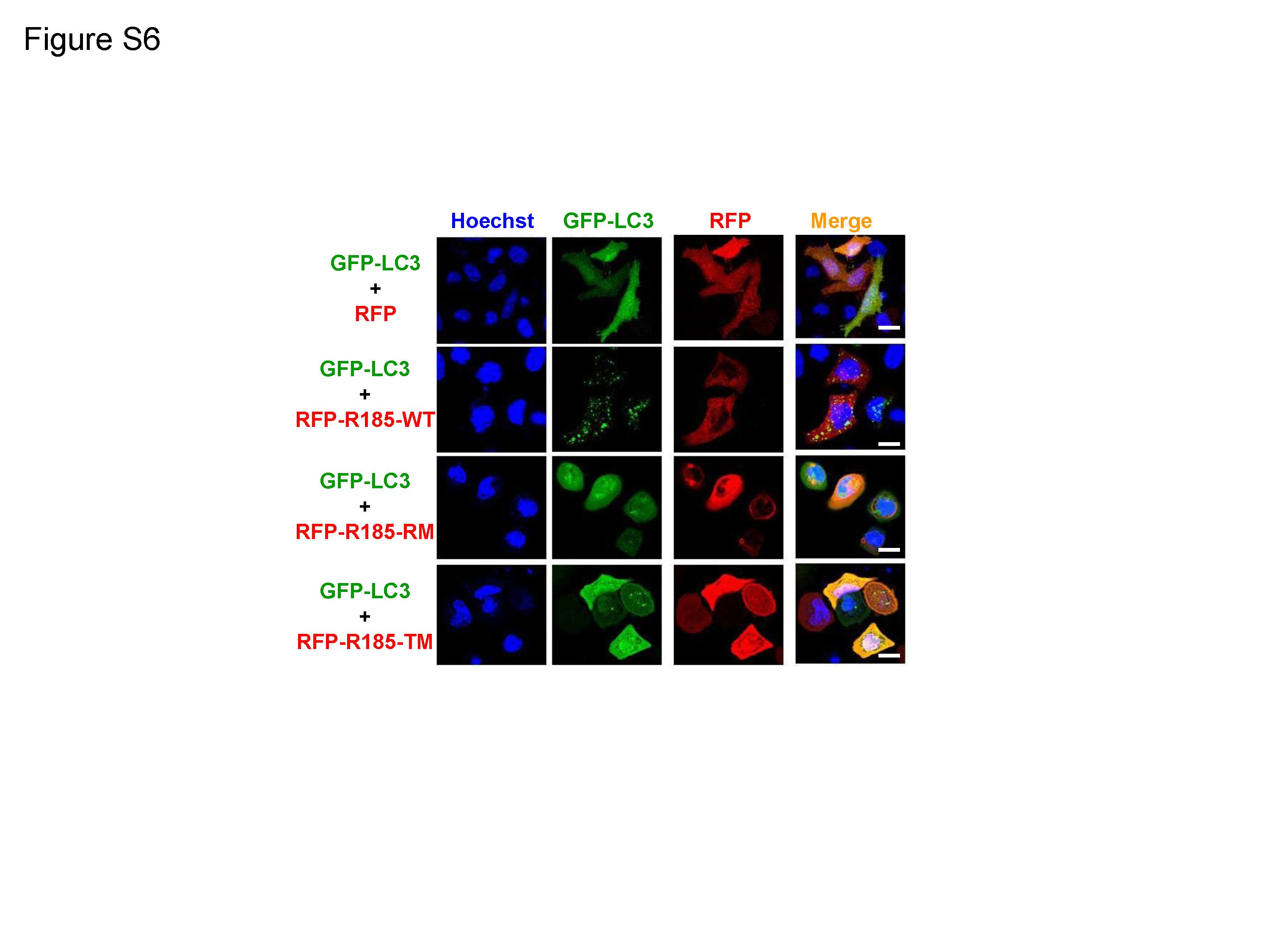

Supplement: Figure S6 — The induction of punctate GFP-LC3 by ectopic expression of RNF185 depends on its RING domain and TM domains. Confocal microscopic analyses were taken at 24 h post the co-transfection of the indicated constructs. WT, wild type; RM, RING domain mutated; TM, transmembrane domains deleted. White bar, 10 µm. (TIF) [file pone.0024367.s006.tif]
