# Supplementary material for: RNF185, a Novel Mitochondrial Ubiquitin E3 Ligase, Regulates Autophagy through Interaction with BNIP1
Source: PLoS One. 2011 Sep 9;6(9):e24367. doi: 10.1371/journal.pone.0024367 (PMC3170314; doi:10.1371/journal.pone.0024367)
Supplement: Table S1 — List of Bcl-2 family proteins with transmembrane domains. (DOC) [file pone.0024367.s007.doc]

**RNF185, a Novel Mitochondrial Ubiquitin E3 Ligase, Regulates Autophagy through Interaction with BNIP1**

**Supplementary Data**

**Table S1.** **List of Bcl-2 family proteins with transmembrane domains.**

| Symbol | GenBank definition | GenBank ID | TM* |
| --- | --- | --- | --- |
| BCL2 | B-cell CLL/lymphoma 2 | BC027258 | C1 |
| BCL-xl | Homo sapiens BCL2-like 1 (BCL2L1), nuclear gene encoding mitochondrial protein | NM_138578 | C1 |
| BCL-w | Homo sapiens BCL2-like 2 (BCL2L2) | BC021198 | C1 |
| MCL1 | myeloid cell leukemia sequence 1 (BCL2-related), BCL2L3 | BC017197 | C1 |
| Boo | BCL2-like 10 (apoptosis facilitator), BCL-B, | NM_020396 | C1 |
| BAX | BCL2-associated X protein, Bcl-2-like protein 4 | BC014175 | C1 |
| BNIP1 | BCL2/adenovirus E1B 19kDa interacting protein 1 | BC010959 | C1 |
| BNIP3 | BCL2/adenovirus E1B 19kDa interacting protein 3 | BC021989 | C1 |
| BNIP3L | BCL2/adenovirus E1B 19kDa interacting protein 3-like, Nix | BC001559 | C1 |
| BIK | BCL2-interacting killer (apoptosis-inducing) | BC001599 | C1 |
| HRK | harakiri, BCL2 interacting protein (contains only BH3 domain) | NM_003806 | C1 |
| BAK1 | BCL2-antagonist/killer 1, Apoptosis regulator BAK, BCL2L7 | BC004431 | C1 |
| BCAP31 | B-cell receptor-associated protein 31 | BC065292 | N3 |
| BCL2L13 | BCL2-like 13 (apoptosis facilitator), Bcl-rambo, Protein Mil1 | BC007658 | C1 |
| TMBIM1 | transmembrane BAX inhibitor motif containing 1, Protein RECS1 homolog | BC026348 | C7 |
| TMBIM2 | Fas apoptotic inhibitory molecule 2(FAIM2), Transmembrane BAX inhibitor motif-containing protein 2 | BC000051 | C7 |
| TMBIM3 | glutamate receptor, ionotropic, N-methyl D-asparate-associated protein 1 (glutamate binding)(GRINA) | BC041788 | C7 |
| TMBIM4 | transmembrane BAX inhibitor motif containing 4, CGI-119, GAAP, Protein S1R | NM_016056 | C7 |
| TMBIM5 | growth hormone inducible transmembrane protein(GHITM), Transmembrane BAX inhibitor motif-containing protein 5 | NM_014394 | C6 |
| TMBIM6 | transmembrane BAX inhibitor motif containing 6, Bax inhibitor 1 , Testis-enhanced gene transcript protein(TEGT) | NM_003217 | N6 |

*TM, position and number for transmembrane domain(s); C1, one TM domain at the C-terminus; N3, three TM domains at the N-terminus. TM domains were predicted by the internet server SMART ( http://smart.embl-heidelberg.de/ ).
